# Should Multifocal Intraocular Lenses Become a Standard in Phacoemulsification Cataract Surgery?

**DOI:** 10.3390/jcm12051983

**Published:** 2023-03-02

**Authors:** Achia Nemet, Piotr Kanclerz, Raimo Tuuminen

**Affiliations:** 1 Department of Ophthalmology, Assuta Ashdod University Medical Center, Ashdod 7747629, Israel; 2 Helsinki Retina Research Group, University of Helsinki, 00290 Helsinki, Finland; 3 Hygeia Clinic, 80-286 Gdańsk, Poland; 4 Department of Ophthalmology, Kymenlaakso Central Hospital, Kotka Hygeia Clinic, 48210 Gdańsk, Finland

Cataracts impair daily activities such as reading, outdoor sports, and driving, which may not match best-corrected visual acuity at optimal room light conditions, but can be just as important to patients [1]. Several anti-cataract drugs were applied in the clinic, but their effects are not still satisfactory [2]. Thus, surgery is the only curative treatment for cataracts. Although cataract surgery is regarded as a low-risk procedure [3], the surgery is often performed on older patients, and various systemic and ocular comorbidities in addition to combined ophthalmic surgeries increase the intra- and postoperative complication rates of the procedure [4,5]. 

Recent developments in several fields such as fluidics, microincisional surgery, and patient stratification have led to improved outcomes and a decrease in complication rates. In this issue, Luo et al. reported that the active-fluidics system enables surgeons to pre-set a target intraocular pressure (IOP) level, and it replenishes the fluids proactively; thus, the IOP is consistently maintained near the target level [6]. A reduction in the complication rates was noted with increasing surgeon seniority, but also with improvements, especially in surgical education and equipment [7]. Thus, the rationale for routine ophthalmic check-ups in low-risk patients have been questioned [8,9]. 

Applying povidone-iodine to the ocular surface is a common part of the preparation of the eye in ophthalmic surgery, and intracameral antibiotics appear to diminish endophthalmitis rates effectively, with or without topical antibiotics additionally [10,11,12]. A significant postoperative complication is pseudophakic cystoid macular edema (PCME), an inflammatory process that may deteriorate visual recovery [13,14]. Most of the PCME cases exhibit the spontaneous resolution of edema within few months, whereas some may develop the chronic refractile form of PCME [14]. Topical corticosteroids and non-steroidal anti-inflammatory drugs (NSAIDs) are prescribed against postoperative inflammation, pain, and the development of PCME [15,16,17]. The use of intravitreal corticosteroids or anti-vascular endothelial growth factor (anti-VEGF) agents has been examined in high-risk cases, as well as when topical medications either fail or have a limited effect [18,19]. In addition, dropless cataract surgery may be a feasible alternative, especially for patients with a poor adherence to eye drops [20,21]. Another common postoperative problem following cataract surgery is aggravation of the symptoms of dry eye, which is common in elderly patients. Jing et al. evaluated the change patterns in corneal intrinsic aberrations and nerve density after cataract surgery in dry eye disease. The authors found that the corneal vortical nerve maximum length and average density negatively correlated with the anterior corneal surface aberrations before and one month after cataract surgery, and that the corneal vortex provided a unique site to observe long-term corneal nerve injury related to eye dryness [22].

Delayed complications of cataract surgery involve retinal detachment, the risk of which seems to be increased for up to 20 years after cataract surgery [23]. In a study of 9400 patients, the cumulative rate of retinal detachment was 2.3% at eight years, and the risk of detachment was evident, especially in highly myopic eyes [24,25]. Posterior capsule opacification (PCO) caused by lens epithelial cell migration, proliferation, and epithelial-mesenchymal transition (EMT) may result in visual symptoms, particularly when involving the central visual axis. A younger age is one of the known risk factors for PCO [26]. Other significant risk factors include myopia (implantation of low-diopter IOLs) and specific IOL properties [27,28]. Furthermore, earlier studies highlight that patients who underwent cataract surgery seem to be at an increased risk for age-related macular degeneration and glaucoma [29,30,31,32]. The suspected mechanisms include increased post-procedure light damage, secondary inflammatory changes, or the induction of angiogenesis. It remains controversial whether blue-light filtration in IOLs may counteract the suspected risk derived from surgery [1]. 

In many cases, cataract surgery visual quality and function outcomes might depend on the presence of ocular comorbidities such as underlying age-related macular degeneration, amblyopia, diabetic retinopathy, glaucoma, and uveitis [33]. Young cataract patients and those who undergo refractive lens exchange (RLE) might have a better self-assessed outcome than elderly patients, with surgical complications and poor near vision correlating with a poor self-assessed outcome [34]. The accuracy of postoperative predicted refraction is constantly improving, with newer-generation IOL power calculation formulae [35]. Still, in complicated cases, the outcome might not be satisfactory. For example, in their recent systematic review, Yahalomi et al. found that in patients with advanced keratoconus (KC), a few of the eyes achieved spherical equivalent refraction within 1 diopter from the target, and the Kane’s formula with keratoconus adjustment showed the best results in all KC stages [36]. This is far from what we could expect in patients with regular corneas. Moreover, the optimal treatment in eyes with insufficient capsular support is still to be determined. In a retrospective study of 28 patients, Franco et al. reported that secondary IOL implantation resulted in similar visual and surgical outcomes between a sutureless Carlevale lens scleral fixation and a suture-free scleral fixation three-piece IOL [37]. Despite excellent outcomes in most of the cases, devasting complications are still reported. Rosen and Vernon documented unexpected poor vision within 24 h of uneventful cataract surgery. Complications are inherently rare in this period; however, various optical, anterior segment, lens-related, and posterior segment causes have been identified, and paracentral acute middle maculopathy (PAMM) remains the only cause of unexpected visual loss within this time frame that may show no abnormal findings on clinical examination [38].

As cataract surgery enables obtaining excellent uncorrected distance visual acuity, the focus of research has shifted towards complete spectacle independence [1]. Current multifocal and extended depth-of-focus (EDOF) IOL designs improve not only vision-related quality of life (VRQoL), but also general health-related quality of life [39]. Both multifocal and EDOF IOLs seem to provide high rates of spectacle independence and patient satisfaction [40,41]. In a study by Shin et al., the clinical outcomes of bilateral implantation between diffractive trifocal IOLs and EDOF IOLs in Koreans emphasized that trifocal IOLs provided near-visual acuity over the EDOF IOLs, whereas intermediate and distance visual acuity were excellent in both types of IOL [42]. Moshirfar et al. showed that in comparison to the visual and refractive performance of two multifocal IOLs, the TECNIS Synergy provided a significantly better uncorrected near visual acuity compared to the AcrySof IQ PanOptix at three and six months postoperatively, whereas patients implanted with TECNIS Synergy reported more early photic phenomena than the patients with Acrysof IQ PanOptix [43]. Importantly, another paper by Gil et al. comparing the contrast sensitivity and quality of vision of patients bilaterally implanted with six different presbyopia-correcting intraocular lenses reported that up to 40–50% of patients implanted with MIOLs reported glare and halos [44]. The growing body of evidence will determine whether multifocal or EDOF IOLs will become a standard in phacoemulsification cataract surgery. The idea of obtaining complete spectacle independence in all patients might be the holy grail of cataract surgery, but, certainly, this concept is more than appealing.
